# Functionalized Prion-Inspired Amyloids for Biosensor Applications

**DOI:** 10.1021/acs.biomac.1c00222

**Published:** 2021-07-01

**Authors:** Marta Díaz-Caballero, Susanna Navarro, Salvador Ventura

**Affiliations:** Institut de Biotecnologia i de Biomedicina and Departament de Bioquímica i Biologia Molecular, Universitat Autònoma de Barcelona, 08193 Bellaterra (Barcelona), Spain

## Abstract

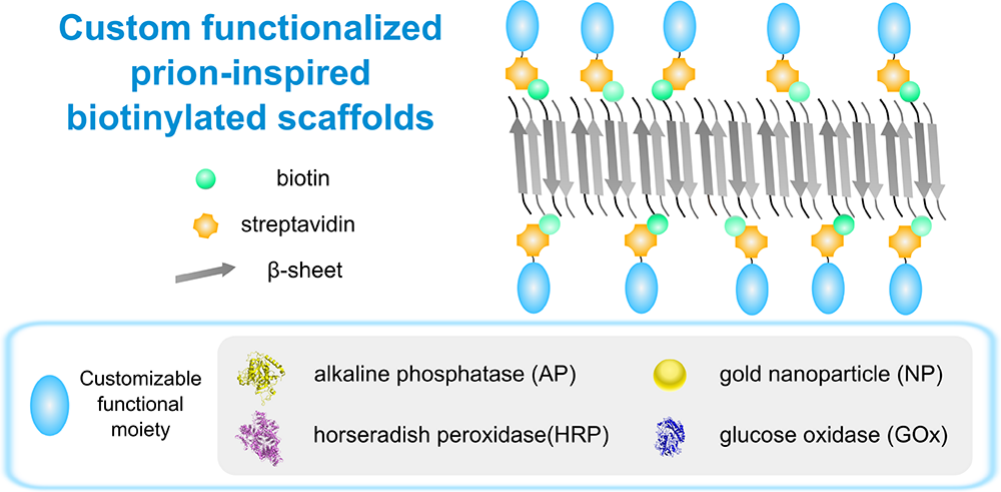

Protein amyloid nanofibers provide a biocompatible platform for the development of functional nanomaterials. However, the functionalities generated up to date are still limited. Typical building blocks correspond to aggregation-prone proteins and peptides, which must be modified by complex and expensive reactions post-assembly. There is high interest in researching alternative strategies to tailor amyloid-based nanostructures’ functionality on demand. In the present study, the biotin-streptavidin system was exploited for this purpose. Prion-inspired heptapeptides (Ac-NYNYNYN-NH_2_, Ac-QYQYQYQ-NH_2_, and Ac-SYSYSYS-NH_2_) were doped with biotin-conjugated counterparts and assembled into amyloid-like fibers under mild conditions. The scaffolds’ versatile functionalization was demonstrated by decorating them with different streptavidin conjugates, including gold nanoparticles, quantum dots, and enzymes. In particular, they were functionalized with peroxidase or phosphatase activities using streptavidin conjugated with horseradish peroxidase and alkaline phosphatase, respectively. Modification of amyloid-like nanostructures has generally been restricted to the addition of a single protein moiety. We functionalized the fibrils simultaneously with glucose oxidase and horseradish peroxidase, coupling these activities to build up a nanostructured glucose biosensor. Overall, we present a simple, modular, and multivalent approach for developing amyloid-based nanomaterials functionalized with any desired combination of chemical and biological moieties.

## 1 Introduction

Molecular self-assembly is a leading bottom-up strategy for fabricating novel complex nanostructures.^1−3^ This approach exploits the selective recognition between molecular building units to fabricate novel architectures with nanometric dimensions. Bionanomaterials, such as protein nanofibers, offer significant advantages over inorganic assemblies since they provide a cost-effective and environmentally friendly way to produce versatile biocompatible nanomaterials.^4,5^ Among them, amyloid fibrils have been gathering considerable attention as biocompatible functional materials^3,6−9^ and for their application as scaffolds in tissue engineering.^10,11^ They are held by densely packed, hydrogen-bonded β-sheets,^12^ and this distinctive supramolecular configuration endows them with a rigid internal order that results in nanostructures with high strength, stability, and high-morphological aspect ratios,^13^ which, together with their insolubility in aqueous media, make them optimal materials for bionanotechnological applications.^3,14^

Many proteins can form amyloids under appropriate experimental conditions.^15,16^ This sequences’ amyloidogenic potential is concentrated in defined short segments,^17−19^ and peptides corresponding to these regions self-assemble autonomously into amyloids;^20^ thus, short peptides, rather than full-length proteins, are the preferred building blocks in nanotechnology.^4^ In this context, we have recently described a new class of binary patterned heptapeptides whose compositions were inspired by those of prion-like domains (Ac-NYNYNYN-NH_2_, Ac-QYQYQYQ-NH_2_, and Ac-SYSYSYS-NH_2_). These synthetic short prion-inspired peptides fully recapitulate the slow hierarchical amyloid self-assembly of intrinsically disordered prion-like domains.^21^ However, their synthesis is much simpler and cheaper than the recombinant production and purification of the long natural sequences, and it is fully scalable. The peptides self-assemble spontaneously, forming regular steric zippers with an antiparallel β-sheet disposition in which polar residues are packed at the interface between β-sheets, yielding a strong network of hydrogen bonds that stabilize the inner amyloid architecture, whereas in the surface of the steric zipper, Tyr residues form ladders of aromatic–aromatic interactions.^22^ Thus, in the resulting nanofibers, every side chain contributes to the structure and stability. In contrast to inorganic nanoparticles or carbon nanotubes, these prion-inspired biocompatible nanostructures are formed under mild experimental conditions, such as room temperature, neutral pH, and ambient pressure.

The ability to decorate peptide nanofibers with functional groups is of paramount importance to utilize them as sensors,^5^ drug delivery systems,^23,24^ or cell scaffolds,^25,26^ among other applications. Fiber post-assembly functionalization is challenging, especially when folded globular proteins should be chemically attached to preformed nanofibers because of the limited availability and expensive polypeptide conjugation chemistry, cross-reactivity, the lack of stoichiometry control, and the unavoidable reduction in the proportion of conformationally active molecules in the assembly.^27^ Therefore, there is a need for alternative methods that allow developing hybrid nanoarchitectures decorated with intact and active chemical and biological moieties in a stoichiometrically controlled manner. In this regard, the use of noncovalent interactions with high affinity and specificity to functionalize nanostructures is gaining momentum.^27−29^

The biotin-streptavidin system provides the strongest noncovalent interaction in nature (*K*_d_ ≈ 10^–14^–10^–16^ M), being exceptionally stable against physical and chemical perturbations, including heating, the presence of denaturant agents or detergents, or extensive washing steps.^30^ In this system, the first component is biotin, also known as vitamin H, a small 245 Da molecule acting as a cofactor in many enzymatic reactions. The second molecule corresponds to streptavidin, a 52 kDa β-barrel protein homotetramer that binds four biotins per monomer. The particular properties of this noncovalent interaction have been exploited for various applications such as immunodetection,^31^ affinity chromatography,^32^ imaging,^33,34^ synthetic chemistry,^35^ chemical biology,^36^ and therapeutics.^37^ This is facilitated by a large number of commercially available streptavidin conjugates, including enzymes, antibodies, and different inorganic molecules.^38^

The avidin/neutravidin/streptavidin-biotin interaction has been utilized for the decoration of peptide nanostructures with fluorescent quantum dots,^39^ enzymes,^31,40,41^ and metallic nanoparticles^42,43^ and exploited for the generation of fibril-metallic hybrid nanowires,^44^ the development of biosensors,^45^ or the design of immunodetection systems.^31,46^ In many of these applications, the nanofibers require post-assembly modifications to conjugate them to biotin and incorporate a single functional component. Here, we have exploited the slow hierarchical self-assembly of prion-inspired heptapeptides to dope them with biotinylated counterparts, obtaining amyloid-like nanofibers that incorporate biotin moieties without a need for any additional manipulation. Using streptavidin conjugates, these nanostructures were functionalized with quantum dots, gold nanoparticles, and enzymes like horseradish peroxidase or alkaline phosphatase. Importantly, this modular strategy allows obtaining multivalent nanofibers, anchoring simultaneously two different catalytic entities, and allowing them to perform coupled enzymatic reactions and act as solid biosensing platforms.

## 2 Experimental Methods

### 2.1 Peptide Preparation

Synthetic heptapeptides NY7 (Ac-NYNYNYN-NH_2_), biotinylated NY7 (Biotin-APAANYNYNYN-NH_2_), QY7 (Ac-QYQYQYQ-NH_2_), biotinylated QY7 (Biotin-APAAQYQYQYQ-NH_2_), SY7 (Ac-SYSYSYS-NH_2_), and biotinylated SY7 (Biotin-APAASYSYSYS-NH_2_) were purchased from CASLO ApS (Scion Denmark Technical University). Lyophilized peptides were dissolved in 1,1,1,3,3,3-hexafluoro-2-propanol (HFIP) to obtain a 10 mM stock solution for NY7, QY7, and SY7 peptides and 1 mM stock solution for biotinylated NY7, QY7, and SY7 peptides, aliquoted and frozen at −80 °C until their use.

### 2.2 Peptide Self-Assembly

Peptides were mixed in a 1:5 (biotinylated peptide:nonbiotinylated peptide) molar ratio to a final concentration of 250 μM (accounting for both peptides) in 100 mM K_2_HPO_4_/KH_2_PO_4_ at pH 7.0. The peptides were diluted directly from HFIP stock solutions. HFIP was 2.5% (v/v) in the final solutions. Self-assembling reactions were performed at room temperature for 7 days under quiescent conditions.

### 2.3 Quantification of Biotinylated and Nonbiotinylated NY7, QY7, and SY7 Assemblies

After 7 day incubation of the reactions under the described conditions, the soluble and insoluble fractions were separated by centrifugation at 12,000*g* for 30 min at room temperature. Supernatant and pellets were preserved for their quantification. Self-assembled peptides corresponding to the insoluble fraction were diluted in 1:10 final volume and incubated with 2.5 M guanidinium thiocyanate (GITC) in 100 mM K_2_HPO_4_/KH_2_PO_4_ buffer at pH 7.0 for a minimum of 12 h under soft agitation to equilibrate the reaction. Calibration curves were obtained using a known concentration of biotinylated and nonbiotinylated peptides ranging from 0 to 50 μM incubated under the same conditions. Tyrosine intrinsic fluorescence was recorded on a Jasco FP-8200 fluorescence spectrophotometer (Jasco Corporation, Japan) in the range 280–400 nm, excited at a 268 nm wavelength, using an excitation and emission bandwidth of 5 nm at 25 °C to determine the concentration of peptides present in each fraction. Fluorescence emission at 303 nm, which corresponds to tyrosine emission maximum, was used to quantify the peptides.

### 2.4 Thioflavin-T binding

Thioflavin-T (Th-T) was employed to determine the presence of amyloid structures in the reactions. Incubated peptides were diluted 1:10 in 100 mM K_2_HPO_4_/KH_2_PO_4_ at pH 7.0, and Th-T was added to a final concentration of 25 μM. Th-T emission fluorescence was detected on a Jasco FP-8200 fluorescence spectrophotometer (Jasco Corporation, Japan) in the range 460–600 nm using an excitation wavelength of 445 nm and with excitation and emission bandwidth of 5 nm.

### 2.5 Light Scattering

Light scattering was measured in a Jasco FP-8200 fluorescence spectrophotometer (Jasco Corporation, Japan), which was excited at 330 nm, and recorded in the range 320–340 nm using an excitation and emission bandwidth of 5 nm.

### 2.6 Fourier Transform Infrared (FTIR) Spectroscopy

Nonbiotinylated and biotinylated peptides were incubated for 7 days as described above, centrifuged at 12,000*g* for 30 min and resuspended in MilliQ water. Samples were placed on the ATR crystal and dried with nitrogen flow. The experiments were carried out in a Bruker Tensor 27 FTIR (Bruker Optics, United States) supplied with a Specac Golden Gate MKII ATR accessory. Each spectrum consists of 32 acquisitions measured at a resolution of 1 cm^–1^. Data were acquired and normalized using the OPUS MIR Tensor 27 software (Bruker Optics, United States). IR spectra were fitted employing a nonlinear peak-fitting equation using PeakFit package v4.12 (Systat Software, San Jose, CA). The area for each Gaussian curve was calculated in the amide I region from 1700 to 1600 cm^–1^ using the second derivative deconvolution method in the PeakFit package v4.12 (Systat Software, San Jose, CA).

### 2.7 Determination of Critical Aggregation Concentration (CAC) by Th-T Binding

Nonbiotinylated and biotinylated peptide reactions were prepared at different concentrations ranging from 0 to 100 μM in 100 mM K_2_HPO_4_/KH_2_PO_4_ at pH 7.0. After 7 day incubation at room temperature, assemblies were diluted to 10 μM in 100 mM K_2_HPO_4_/KH_2_PO_4_ at pH 7.0, and Th-T was added to a final concentration of 5 μM. The absorbance values at 492 nm were employed to plot the Th-T fluorescence signal versus the peptide concentration. Linear regressions of Th-T fluorescence intensities and the basal Th-T signal were calculated using GraphPad Prism 6.0. Two different regressions were calculated: the first one corresponding to low concentration reactions (between 0 and 10 μM) with similar intensities to that of the Th-T basal signal and the second one corresponding to concentrations ranging from 25 to 100 μM with higher Th-T intensity. The intersection of the two linear regressions corresponds to the critical aggregation concentration (CAC) for each reaction. Measurements were acquired in triplicates, and the mean and standard deviation were calculated.

### 2.8 Streptavidin-Gold Nanoparticles (Gold NPs) Binding to Biotinylated Scaffolds

A total of 100 μL of NY7, QY7, and SY7 biotinylated fibers was centrifuged at 12,000*g* for 30 min. The pellet was resuspended in 50 μL of MilliQ water. Streptavidin-gold NP conjugate solution (Sigma Aldrich, Germany) was diluted 1:10 (v/v) in MilliQ H_2_O and equilibrated at room temperature for 20 min. After equilibration, 50 μL of biotin-containing fibers and 50 μL of diluted streptavidin-gold NP solution were mixed to a final dilution 1:20 of streptavidin-gold NPs, and the solutions were incubated overnight at room temperature with soft agitation. Samples were washed twice by centrifugation at 12,000*g* for 30 min and resuspension in MilliQ water to remove the excess of NPs. Samples were deposited onto carbon-coated copper grids for 10 min and stained with 0.5% uranyl acetate solution for 30 s for their visualization by transmission electron microscopy (TEM).

### 2.9 Inductively Coupled Plasma-Atomic Optic Emission Spectrometry (ICP-OES) for Gold Content Quantification

Biotinylated NY7, QY7, and SY7 fibrils and control nonbiotinylated NY7, QY7, and SY7 fibers were washed and incubated in the presence of streptavidin-gold NP conjugate solution (Sigma Aldrich, Germany) diluted 1:20 (v/v) in MilliQ water overnight at room temperature under agitation. Samples were washed twice by centrifugation at 12,000*g* for 30 min and resuspension in MilliQ water to remove the excess of NP. Since some NPs could still precipitate nonspecifically with the fibrils, incubated nonbiotinylated fibrils were used as negative controls. Pellets were stored for their subsequent analysis. Pelleted fibers were resuspended in a HNO_3_ and HCl solution in a ratio 1:3 and heated in a DINKO D-65 heating block to promote peptide digestion. Digestion products were injected in an inductively coupled plasma-optic emission Optima 4300DV mass spectrometer (PerkinElmer, United States) to quantify Au content. The amount of oxidized Au was calculated relative to that of initial fibrils. All samples were measured in duplicates, and the standard deviation was calculated. Significance was calculated with a one-way ANOVA statistical test.

### 2.10 Transmission Electron Microscopy

Grids were exhaustively scanned using a JEM 1400 transmission electron microscope (JEOL Ltd., Japan) operating at 80 kV, and images were acquired with a CCD GATAN ES1000W Erlangshen camera (Gatan Inc., United States).

### 2.11 Quantification of Gold NPs

TEM micrographs were employed to calculate the number of gold NPs visualized in the samples. Image J Software (NIH, United States) was used to calculate the number of gold NPs per μm^2^. We employed at least five micrographs from two different experiments to analyze the retention capacity. Statistical analysis was performed by a one-way ANOVA test followed by a Bonferroni’s Multiple comparison test.

### 2.12 Dot Blotting Streptavidin-Horseradish Peroxidase Coupling to Biotin Scaffolds

Biotinylated NY7, QY7, and SY7 scaffolds were centrifuged at 12,000*g* for 30 min. The pellet was resuspended in the same volume of 100 mM K_2_HPO_4_/KH_2_PO_4_ at pH 7.0. Isolated biotinylated scaffolds were deposited onto a PVDF membrane (Immobilon-P Transfer Membranes, Millipore Corporation, United States) in different amounts (1 and 5 μL), and drops were let dry at room temperature. Nonbiotinylated NY7, QY7, and SY7 scaffolds were used as a negative control in the same amounts as biotin-containing fibers, and biotinylated NY7, QY7, and SY7 soluble peptides at 60 μM were used as positive controls, adding 5 μL. After sample deposition, the membrane was blocked with Blocking Solution (5% w/v Milk in 1× Tween20 Tris buffered saline (TTBS 1×) buffer) for 1 h at room temperature. After blocking, it was incubated with streptavidin-HRP (Abcam, United Kingdom) diluted 1:5000 in 5% (w/v) Milk TTBS 1× solution overnight at 4 °C. The membrane was washed three times with TTBS 1× buffer for 10 min every washing step. Development of the membrane was performed with an Immobilon Forte Western HRP substrate (Millipore Corporation, United States) in a VersaDoc Imaging device (Bio-Rad, United States) using the blotting application with Chemi Ultra sensitivity, clear 0.5× gain and 4 × 4 Bin. The exposure for the acquisition of images was 10 s.

### 2.13 Dot Blotting Streptavidin-Alkaline Phosphatase Coupling to Biotin Scaffolds

NY7, QY7, and SY7 fibers were centrifuged at 12,000*g* for 30 min. The pellet was resuspended in the same volume of 100 mM K_2_HPO_4_/KH_2_PO_4_ at pH 7.0. A total of 50 μL of isolated biotinylated and nonbiotinylated fibers were deposited onto a PVDF membrane (Immobilon-P Transfer Membranes, Millipore Corporation, United States), and drops were let dry at room temperature. A total of 50 μL of each biotinylated NY7, QY7, and SY7 non-assembled peptide at 60 μM was used as controls. After sample deposition, the membrane was blocked with Blocking Solution (5% w/v Milk in 1× Tween20 Tris buffered saline (TTBS 1×) buffer) for 1 h at room temperature. After blocking, it was incubated with streptavidin-AP (Invitrogen, United States) diluted 1:1000 in 5% (w/v) Milk TTBS 1× solution overnight at 4 °C. The membrane was washed three times with TTBS 1× buffer for 15 min every washing step. Development of the membrane was performed with a Check BCIP/NBT Liquid Substrate System (Sigma Aldrich, Germany) for 3 h.

### 2.14 Streptavidin-Alkaline Phosphatase Binding to Biotinylated Scaffolds

After 7 days of incubation, NY7, QY7, and SY7 biotinylated peptide samples were centrifuged at 12,000*g* for 30 min to isolate fibrils and the pellet was resuspended in the same volume of 100 mM K_2_HPO_4_/KH_2_PO_4_ at pH 7.0. Streptavidin-AP solution (1 mg/mL, Invitrogen, United States) was added to the biotin-containing scaffolds at a 1:120 ratio (biotin:streptavidin-AP) and incubated overnight at room temperature with soft agitation. Samples were then centrifuged at 12,000*g* for 30 min, and pellets were resuspended in four times the initial volume of 100 mM K_2_HPO_4_/KH_2_PO_4_ at pH 7.0.

### 2.15 Alkaline Phosphatase Activity Assay

Kinetics of AP activity were performed using one-step pNPP (*p*-nitrophenyl phosphate) solution (Thermo Fisher, United States), which was transformed to *p*-nitrophenol (pNP), developing yellow color and an absorbance maximum at 405 nm. This assay was performed in a 96-well plate with the corresponding streptavidin-AP enzyme controls. A total of 10 μL of sample and 90 μL of pNPP solution were mixed. The reaction was stopped by adding 50 μL of 2 M NaOH at corresponding time points. Selected time points were 0, 2, 5, 10, 15, 20, 25, 30, 40, 50, and 60 min. Enzyme control was performed with the same amount of streptavidin-AP added to the biotin-containing scaffold solution (1:120 dilution) and diluted four times just before the addition into the well. Absorbance was measured at 405 nm in a Victor3 (PerkinElmer, United States) plate reader. Experiments were performed in triplicates, results correspond to the mean of two independent experiments, and errors correspond to the standard deviation.

### 2.16 Glucose Oxidase and Horseradish Peroxidase Coupled Reaction Activity Assay

After 7 day incubation, 500 μL of aggregated reactions at 250 μM was centrifuged at 12,000*g* for 30 min to isolate fibers and pellets were resuspended in the same volume of 100 mM K_2_HPO_4_/KH_2_PO_4_ at pH 7.0. Streptavidin-GOx solution (1 mg/mL, Stereospecific Detection Technologies GmbH, Germany) and streptavidin-HRP solution (1 mg/mL, Abcam, United Kingdom) were added to the biotin-containing scaffolds in a 1:1 molar ratio (GOx:HRP) at a final streptavidin-enzyme concentration of 50 nM. Samples were incubated overnight at room temperature with soft agitation. Then, samples were centrifuged at 12,000*g* for 30 min, and pellets were resuspended in eight times the initial volume of 100 mM K_2_HPO_4_/KH_2_PO_4_ at pH 7.0. For non-streptavidin-bound GOx-containing reaction, biotinylated peptides were incubated in the presence of 50 nM streptavidin-HRP conjugate and washed twice. Just before starting the reaction, 50 nM soluble glucose oxidase (GOx) (Sigma Aldrich, Germany) was added to the biotinylated self-assembled samples. Kinetics of GOx-HRP coupled reaction were performed using d-glucose at a final concentration 1 mM (Sigma Aldrich, Germany) and ABTS reagent at a final concentration 2mM (Sigma Aldrich, Germany), which was transformed to ABTS^+^, presenting a green-blue coloration. Reactions were performed in a 96-well plate together with streptavidin-GOx and streptavidin-HRP controls. Kinetics with soluble GOx and streptavidin-HRP and with streptavidin-HRP alone were performed as a positive and negative reaction controls, respectively. For each reaction, 50 μL of sample was added to 50 μL of 4 mM d-glucose and 100 μL of 4 mM ABTS. The absorbance of the product was measured at 405 nm in a Victor3 (PerkinElmer, United States) plate reader, and ABTS^+^ concentration was calculated using ε_405nm_ = 3.65 × 10^4^ M^–1^ cm^–1^. Experiments were performed in triplicates, results correspond to the mean of three independent experiments, and error corresponds to standard deviation.

### 2.17 Streptavidin-Quantum Dots (QD) Binding to Biotinylated Scaffolds

A total of 100 μL of NY7 and biotinylated NY7 fibers at 250 μM was centrifuged at 12,000*g* for 30 min. Pellets were resuspended in PBS-1% BSA and incubated with streptavidin-QD (Thermo Fischer; United States) ranging from 150 to 2000 pM for 30 min at room temperature. To discard the unbound streptavidin-QD, fibers were centrifuged at 12,000*g* for 30 min and the pellets were resuspended in PBS twice. QD fluorescence was measured using a Victor3 Multilabel Reader (PerkinElmer Waltham, MA, USA) with an excitation wavelength 300 nm, an emission wavelength 525 nm, and a bandwidth 20. Visualization of biotinylated NY7 fibers incubated with streptavidin-QD at 1000 pM was performed using a HCX PL APO 63 × 1.4 oil immersion objective on a Leica TCS SP5 microscope (Leica Microsystems, Germany) using excitation at 488 nm and the emission was collected at 515–560 nm with a line average 2.

## 3 Results and Discussion

### 3.1 Biotinylation of Prion-Inspired Heptapeptides

In the design of an amyloid-based biotin-streptavidin system, it was important to avoid steric impairments that might interfere with the self-assembly or reduce the accessibility of streptavidin-conjugated partners to biotinylated peptides once they become embedded into the amyloid fibers. Therefore, a spacer was incorporated between the N-terminal conjugated biotin moiety and the prion-inspired heptapeptides. The short APAA sequence was selected since it has been previously used as a connecting linker between amyloid prone peptides and biotin.^47^ Poly-Ala peptides have been described to form α-helices,^48^ and Pro was incorporated in the second position due to its dual α-helix and β-sheet disruption capacity.^49^ The idea was that the linker would remain disordered and would not interfere with the heptapeptide prion-inspired core’s capability to assemble into a β-sheet structure. Additionally, to avoid the interference of repulsive charges in the self-assembling process, the C-termini of the biotinylated peptides were blocked by amidation.^21^ The final design of the peptide sequences corresponded to Biotin-APAANYNYNYN-NH_2_ (Biotin-NY7), Biotin-APAAQYQYQYQ-NH_2_ (Biotin-QY7), and Biotin-APAASYSYSYS-NH_2_ (Biotin-SY7).

### 3.2 Biotin-NY7, Biotin-QY7, and Biotin-SY7 Peptides Self-Assemble into Amyloid Fibers

Initial experiments were addressed to determine if the amyloid self-assembling capacity of the prion-inspired peptides^21^ was affected by the addition of the biotin molecule and the spacer at the N-terminal edge. Once optimal self-assembly conditions were selected, the peptides were incubated in a 1:5 biotinylated-peptide:nonbiotinylated peptide molar ratio. This doping strategy provides a defined streptavidin binding stoichiometry and avoids possible steric constraints restricting incoming molecules’ access to N-terminal biotins. In addition, if two biotinylated peptides become adjacent in the fiber, the steric zipper’s antiparallel nature^22^ would place their biotin moieties at opposite ends of β-sheets, precluding steric clashing between incoming streptavidin domains. Reactions were prepared at a final peptide concentration of 250 μM in 100 mM K_2_HPO_4_/KH_2_PO_4_ buffer at pH 7.0 and incubated for 7 days at 25 °C under quiescent conditions. From now on, assemblies formed by a 1:5 biotinylated peptide:nonbiotinylated peptide molar ratio will be referred to as biotinylated assemblies, fibers, or scaffolds, unless otherwise indicated.

**Figure 1:**
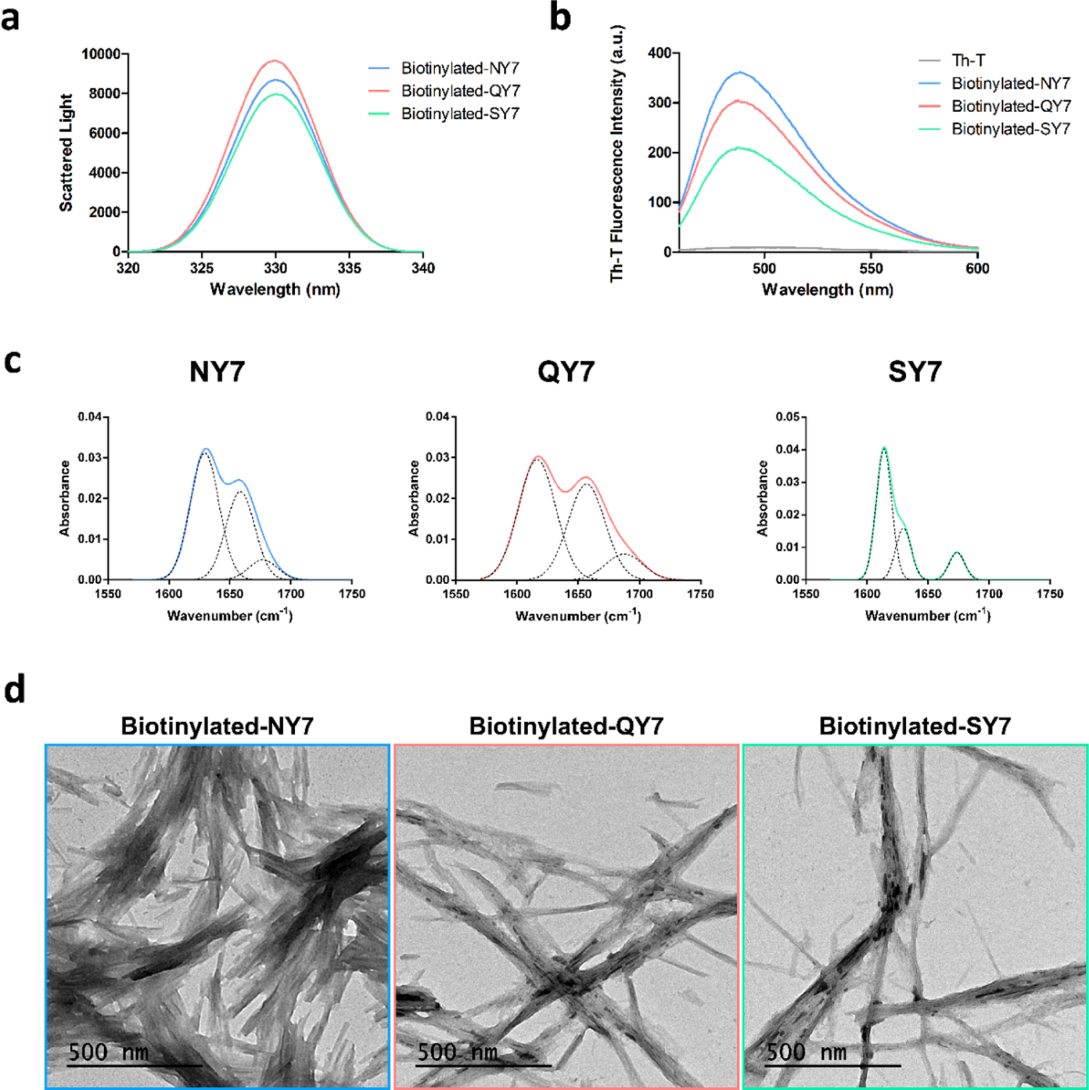
Biophysical characterization of biotinylated NY7, biotinylated QY7, and biotinylated SY7 amyloid scaffolds. Biotinylated NY7 (blue), biotinylated QY7 (red), and biotinylated SY7 (green) peptides were prepared at 250 μM to a 1:5 (biotinylated:nonbiotinylated peptide) ratio in buffer 100 mM K_2_HPO_4_/KH_2_PO_4_ at pH 7.0, and aggregation was analyzed after 7 days of incubation. (a) Synchronous light scattering. (b) Amyloid tinctorial properties of incubated peptides were assessed by Th-T in the absence (gray line) and in the presence of biotin-containing peptides. (c) FTIR absorbance spectra in the amide I region (solid line) of biotinylated NY7 (blue), biotinylated QY7 (red), and biotinylated SY7 (green) assembled peptides. Dashed black lines indicate the different signals contributing to the absorbance spectrum. (d) Morphology characterization by TEM. Scale bars correspond to 500 nm.

The peptide mixture’s ability to self-assemble was first monitored by synchronous light scattering (Figure 1a). A significant scattering signal was recorded for the three incubated peptides, reporting large assemblies in the solution. Then, the amyloid-like nature of these peptide aggregates was assessed using the amyloid binding dye Thioflavin-T (Th-T). All peptide solutions promoted a substantial increase in Th-T fluorescence intensity upon incubation with the dye, compared with Th-T alone (Figure 1b).

The critical aggregation concentration (CAC) is a parameter often used to approach the concentration at which premicellar aggregates are formed. The CAC values for biotinylated and nonbiotinylated assemblies were determined by measuring Th-T fluorescence, as an amyloid-like structure reporter, in the 0.2–100 μM initial peptide concentration range (Figure S1 and Table S1). The CACs of the different nonbiotinylated peptides and of reactions containing a 1:5 ratio of biotinylated:nonbiotinylated peptides did not differ significantly, lying in the 10–20 μM range in all cases.

We used Fourier transform infrared (FTIR) to investigate the secondary structure of NY7, QY7, and SY7 biotinylated assemblies, recording absorbance in the amide I region of the spectrum (Figure 1c). The vibrational contributions were calculated from the Fourier transformed curves (Table S2). The presence of the biotin molecule and the linker did not significantly alter the secondary structure of the biotinylated assemblies relative to the corresponding nonbiotinylated forms (Figure S2) with a β-sheet content higher than 60% in all samples. However, there are important differences in the assemblies’ secondary structure depending on the constituent peptide’s identity, with SY7 generating the more ordered assemblies (Figure 1, Figure S2, and Table S2).

The morphology of biotinylated and nonbiotinylated assemblies was inspected by transmission electron microscopy (TEM). Incubated samples were deposited onto carbon-coated copper grids and negatively stained using uranyl acetate solution. The images’ visualization revealed the presence of long fibers for the three biotin-containing heptapeptides (Figure 1d), analogous in morphology to those observed for the equivalent nonbiotinylated peptides^21^ (Figure S2b), in excellent agreement with their very similar secondary structure, as analyzed by FTIR (Figure S2a).

Overall, the biophysical characterization confirmed that neither biotin conjugation nor the linker at the N-terminus significantly influenced the peptides’ self-assembly into amyloid-like fibrillar structures.

A similar pre-assembly doping approach has been successfully used to build up biotin-decorated fibers of the Alzheimer’s-related Aβ40 peptide.^50^ Compared with the synthetic heptapeptides, Aβ40 fibrillation is more stochastic and challenging to reproduce lab to lab, the building unit is significantly more expensive, and the use of Aβ40 assemblies for biomedical applications is a subject of debate due to their potential cytotoxicity. In contrast, prion-inspired fibrils are inert.^21^ Another successful pre-assembly strategy to decorate amyloid-like fibers with biotin consists of genetically fusing an autobiotinylated peptide to the selected amyloidogenic protein of interest.^31,46^ However, it requires the recombinant expression and posterior purification of a comparatively longer protein fusion, being less straightforward, scalable, and significantly more expensive than the approach that we use here.

### 3.3 Functionalization of Nanofibers with Gold Nanoparticles

**Figure 2:**
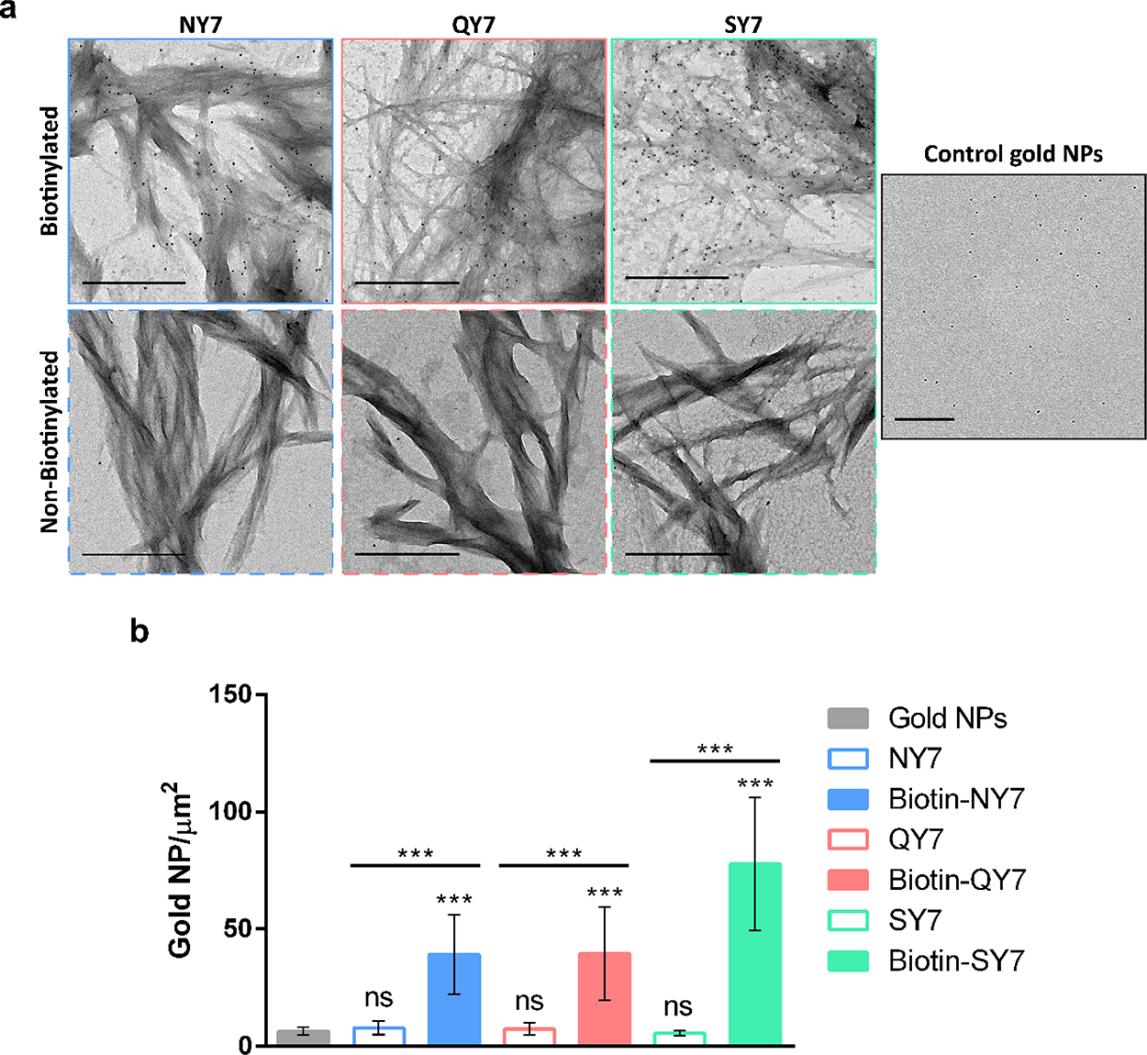
Characterization of biotinylated NY7, biotinylated QY7, and biotinylated SY7 amyloid scaffolds incubated with streptavidin-gold NP conjugates. (a) TEM micrographs of biotinylated NY7 (blue), biotinylated QY7 (red), and biotinylated SY7 (green) peptides were prepared at 250 μM to a 1:5 (biotinylated peptide:nonbiotinylated peptide) ratio, corresponding to the top-line images with continuous lines. Dashed lined images at the bottom correspond to nonbiotinylated scaffold controls. Streptavidin-gold NP conjugate control corresponds to the right image (black continuous line). Scale bars correspond to 500 nm. (b) Quantification of gold NPs. TEM micrographs acquired in panel (A) were used to quantify gold NPs, and the content was expressed as numbers of gold NPs/μm^2^. Data correspond to the average quantification from at least five micrographs from two independent experiments, and bars correspond to the SD of the mean. For all peptides, the one-way ANOVA statistical test was performed relative to the gold NP control (***P-value < 0.001, **P-value < 0.01, and *P-value < 0.05) followed by a Bonferroni’s multiple comparison test (***P-value < 0.001, **P-value < 0.01, and *P-value < 0.05) to compare biotinylated and nonbiotinylated samples.

The binding of streptavidin-gold nanoparticle (NP) conjugates to the biotinylated amyloid scaffolds was visualized by TEM. Micrographs clearly showed small black dots corresponding to gold NPs distributed along the biotinylated fibers’ surface (Figure 2a). In contrast, when the fibers formed by nonbiotinylated peptide counterparts were incubated with streptavidin-gold NPs, such colocalization was not observed, indicating that the presence of biotin within these matrices was a requirement for the incorporation of NPs to the fibers. The presence of some randomly distributed black dots on the grids could be attributed to the gravitational deposition of NPs, as shown in the negative control micrograph in the absence of fibers (Figure 2a).

The number of gold NPs per μm^2^ was quantified in the TEM micrographs (Figure 2b). As a reference, we employed the negative control micrographs (Figure 2a), and they were compared with nonbiotinylated and biotinylated fibers. The number of gold NPs detected in nonbiotinylated fibers was not statistically different from that of the control for the three peptides (Figure 2b). On the contrary, the number of gold NPs in the three biotinylated fibers was higher than those in the negative control and the corresponding nonbiotinylated fibers. The difference in gold NP content for each pair of nonbiotinylated and biotinylated fibers was statistically significant in all cases. However, the comparison between the gold NP content of biotinylated fibrils formed by different peptides is only qualitative since the number of bound nanoparticles depends on the number of peptide monomers and the number of fibrils in the micrographs, which cannot be accurately quantified.

To demonstrate orthogonally the ability of biotinylated fibers to capture gold NPs, we used the inductively coupled plasma-optic emission spectroscopy (ICP-OES) technique. The amount of gold NPs in biotinylated and nonbiotinylated fibril samples was calculated relative to that of fibrils (Table S3). Nonspecific precipitation of gold NPs with fibrils during the washing procedure resulted in the presence of Au in nonbiotinylated samples. However, the quantity of Au in biotinylated fibril samples was significantly higher for all the assayed peptides (*P*-value < 0.01). The specific binding of streptavidin gold NPs to biotinylated scaffolds accounts for ∼20 ng gold NPs/μg fiber.

Gold nanoparticles and other metals have been combined with different bionanostructures to build up conductive nanomaterials.^44,51−53^ In particular, metalized amyloids have been exploited for the generation of nanocables^54^ and nanowires^55,56^ for molecular biosensing using plasmon resonance techniques and for imaging, among other applications.^43,44,51,57^ In this context, the continuous coverage of the prion-inspired scaffold surface with inorganic metallic nanoparticles might allow the implementation of hybrid conductive nanomaterials.

### 3.4 Functionalization of Nanofibers with Enzymatic Activities

Once the biotinylated scaffolds’ capacity to bind streptavidin was demonstrated, we exploited this property to build up biocatalytic systems. The currently employed strategies to generate enzymatically active matrices are often expensive and technically challenging. The traditional techniques for the immobilization of enzymes in solid supports rely on chemical modifications of the catalysts to promote their attachment, a process that might significantly impact their nativeness and activity. Protein engineering advances have allowed the fusion of streptavidin to almost any protein of interest,^34,38,58^ allowing them to decorate biotin-modified matrices. Importantly, in these matrices, a majority of the target protein keeps its native and active conformation. We examined the functionalization of our biotinylated assemblies with two of the most popular enzymes using either streptavidin-horse radish peroxidase (HRP) or streptavidin-alkaline phosphatase (AP) conjugates.

#### 3.4.1 Functionalization of Nanofibers with Peroxidase Activity

**Figure 3:**
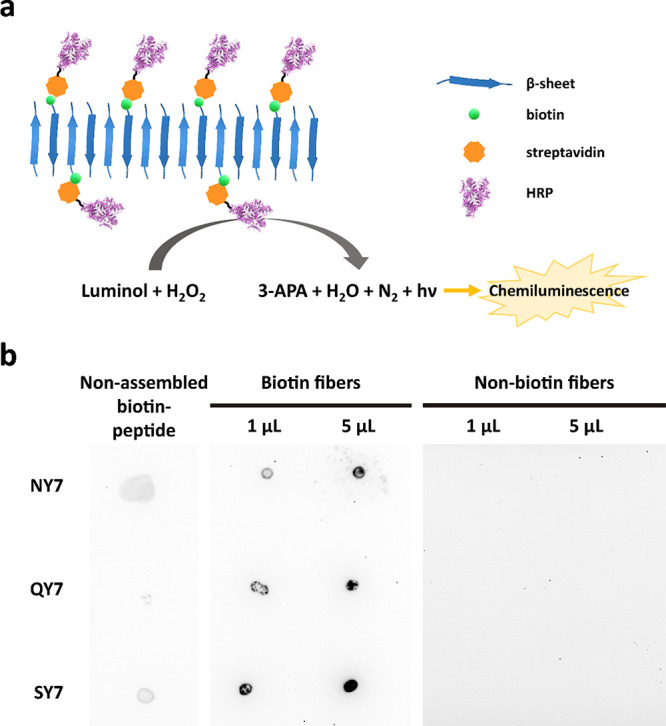
Detection of streptavidin-HRP conjugate activity bound to biotinylated NY7, biotinylated QY7, and biotinylated SY7 amyloid scaffolds. (a) Schematic representation of the reaction catalyzed by HRP within the scaffold. (b) Dot blot of the biotinylated NY7, biotinylated QY7, and biotinylated SY7 scaffolds. Controls correspond to non-assembled biotinylated peptides and to scaffolds without biotin. Different volumes of scaffolds containing solutions were loaded on the membrane: 1 and 5 μL.

The first functionalization assay consisted of the decoration of the biotinylated prion-inspired assemblies with the streptavidin-HRP conjugate. HRP enzyme is employed in multiple detection techniques and commercial kits like Western blotting or ELISA detection assays. It catalyzes a reaction in which the luminol substrate and H_2_O_2_ are transformed into H_2_O, N_2_, and 3-aminophtalate (3-APA), emitting light (Figure 3a). To determine if streptavidin-HRP conjugates could bind specifically to biotinylated fibers, we performed a dot blot assay. The fibers were immobilized onto a membrane and incubated in the presence of streptavidin-HRP conjugate; nonbiotinylated fibers were treated in the same manner as a control. Samples containing biotin displayed a quantity-dependent chemiluminescent signal after incubation with the HRP substrate (Figure 3b). Conversely, fibers without biotin did not exhibit any significant chemiluminescence. Non-assembled biotin-labeled peptides at 60 μM, dissolved in phosphate buffer immediately before their application to the membrane, displayed faint signals. This concentration corresponds to the maximum amount of biotinylated peptide that can be potentially embedded in the scaffolds. Because in this control sample, all peptides bear a biotin moiety that can bind to a streptavidin-HRP unit, a signal at least as strong as the one of the fibrils was expected. The significantly higher chemiluminescent signal in the biotinylated fibers indicates the advantage of concentrating the activity on a reduced surface to increase the sensitivity for this kind of membrane-based assays. It could also be that the mild hydrophobic environment provided by the exposed tyrosine residues in the fibril might be favorable for streptavidin binding.^59^ In any case, it becomes clear that biotinylated prion-inspired assemblies can be effectively functionalized with peroxidase activity.

#### 3.4.2 Functionalization of Nanofibers with Alkaline Phosphatase Activity

**Figure 4:**
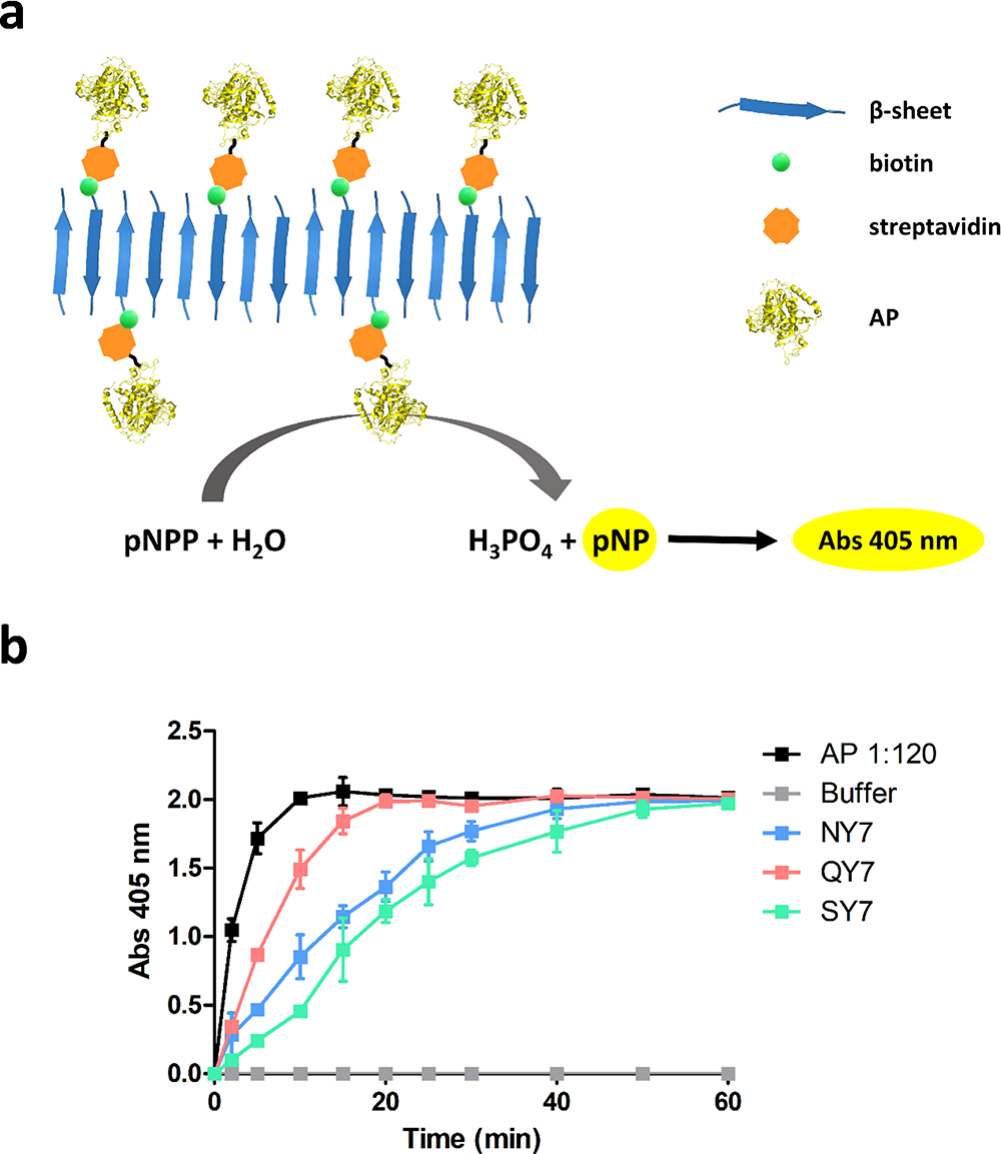
Biotinylated NY7, biotinylated QY7, and biotinylated SY7 amyloid scaffolds functionalized with the streptavidin-AP conjugate. (a) Schematic representation of the reaction catalyzed by AP within the scaffolds. (b) Kinetics of the biotinylated NY7 (blue), biotinylated QY7 (red), and biotinylated SY7 (green) scaffolds bound to streptavidin-AP. Controls correspond to soluble streptavidin-AP (black) and to buffer (gray). Results correspond to the mean of three independent experiments ± standard deviation.

Further on, we addressed the functionalization of prion-inspired amyloid scaffolds with a streptavidin-AP conjugate. AP enzyme’s primary physiological role corresponds to dephosphorylation. This activity has been for many applications, and in particular as a reporter in immunoassays. AP activity has been classically assessed using the *p*-nitrophenyl phosphate (pNPP) substrate, which is hydrolyzed into phosphoric acid and *p*-nitrophenol (pNP). This pNP product presents a yellow coloration at alkali pH (around pH 8.0) with a maximum absorbance peak at 405 nm (Figure 4a).

Biotinylated scaffolds were incubated with streptavidin-AP, and the AP activity was detected by measuring the appearance of the pNP product. The experiment ratified the affinity of these assemblies for streptavidin conjugates (Figure 4b), with all biotinylated fibers displaying significant AP catalytic activity.

The binding of streptavidin-AP conjugates to biotinylated scaffolds was also assessed by a dot blot assay, as previously described for HRP. Biotinylated fibers were immobilized onto a membrane and incubated with the streptavidin-AP conjugate. In this case, we detected the formation of visible NBT formazan. This product is insoluble and is deposited on the membrane at the places where AP is present. In the presence of fibers, we observed dark blue precipitates (Figure S3), whereas a 60 μM solution of non-assembled biotinylated peptides provided a faint blue background (Figure S3), indicating again that the enzymatic activity concentrates on top of the discrete fibrillar aggregates.

The results in this and the previous section suggest that, in principle, our scaffolds can be functionalized with any enzymatic activity of interest, as long as a streptavidin conjugate is available. The approach’s modularity presents advantages compared with previously explored strategies that rely on protein fusions to build up functionalized fibers, whose activity is limited to the one encoded in the fused globular domain.^31,60,61^

### 3.5 Dual Functionalized Nanofibers Allow Glucose Biosensing

Even when an enzyme reaction results in an undetectable product, it can still be possible to detect it using a coupled assay, where the product is used as the substrate of another easily detectable enzymatic reaction.^62^ Indeed, assays in which two or more enzymes are coupled together are popular in clinical biochemistry.

A common approach to enhance operational performance of biocatalytic systems is the immobilization of the enzymes in a solid matrix. Up to date, the matrices and scaffolds employed for enzyme immobilization are mostly inorganic and demand additional chemical modifications of the protein to attach it covalently to the matrix as well as long-time incubation steps,^63−65^ which might compromise the enzyme’s conformation and activities.^65^ This is especially critical when two or more enzymes should be simultaneously bound to the surface since they would differ in their stability, and the optimal conditions for the cross-linking of one protein to the matrix in a native conformation would not necessarily apply to the other/s. In this context, the use of the here described biocompatible amyloid scaffolds conjugated to biotin might significantly reduce the time required for the conjugates’ attachment and prevent the conformational damage and the associated loss of activity. At the same time, it might allow modulating the quantity of enzyme retained in the surfaces according to the available biotin molecules within the matrix. Despite these potential advantages, to the best of our knowledge, coupled reactions of enzymes immobilized into amyloid scaffolds employing the biotin-streptavidin interaction system have not been reported yet.

**Figure 5:**
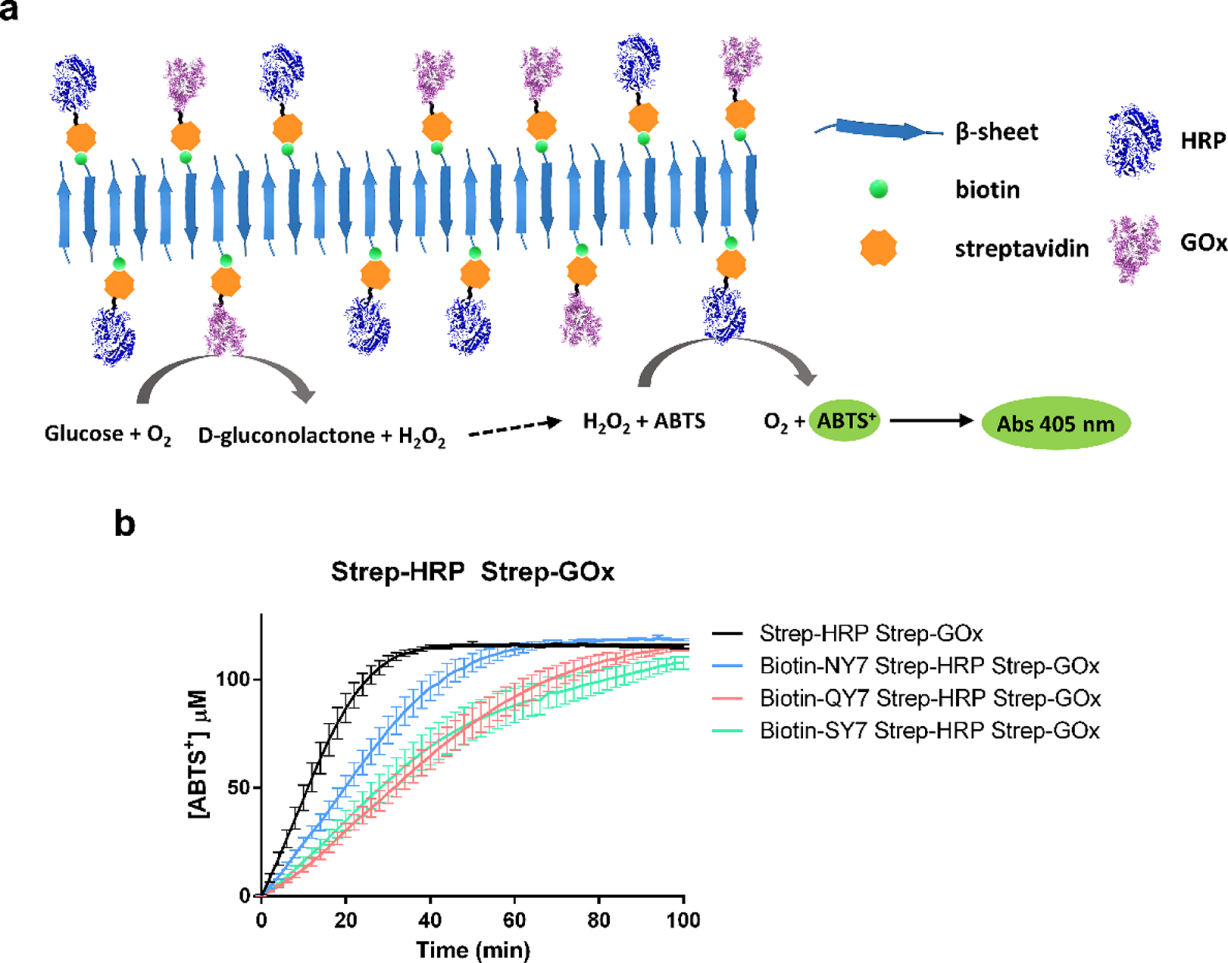
Biotinylated NY7, biotinylated QY7, and biotinylated SY7 amyloid scaffolds functionalized with streptavidin-HRP and with streptavidin-GOx or soluble GOx. (a) Schematic representation of the reaction occurring in the graph below. (b) Biotinylated peptides in the presence of streptavidin-HRP and streptavidin-GOx. Reaction control was streptavidin-GOx and streptavidin-HRP (1 to 1 molar ratio). Results correspond to the mean of three independent experiments ± standard deviation.

We applied our prion-inspired biotinylated fibers to set up a dual-enzyme cascade inspired by glucose sensing.^66^ We immobilized equimolar amounts of glucose oxidase (GOx) and horseradish peroxidase (HRP) simultaneously, exploiting the biotin-streptavidin interaction. In this system, the first reaction corresponds to GOx, which catalyzes glucose’s oxidation into d-gluconolactone and H_2_O_2_. Then, the secondary activity of HRP employs H_2_O_2_ to oxidize ABTS into ABTS^+^, rendering a green-colored product (Figure 5a). Therefore, since there is no H_2_O_2_ in the initial reaction, the HRP activity is only detected in the presence of glucose when the sugar is oxidized by GOx, generating H_2_O_2_ as an intermediate substrate.

Enzyme kinetics revealed a time-dependent increase in absorbance due to ABTS^+^ production, indicating that the incubated amyloid scaffolds bind both streptavidin-GOx and streptavidin-HRP conjugates (Figure 5b). A control with a soluble streptavidin-GOx and streptavidin-HRP mix at the same concentrations at which they were initially added to fibers was performed to know the maximum detectable product. Under the assay conditions, the apparent product concentration of the cascade reaction reaches ∼100 μM ABTS^+^ for all tested scaffolds, being comparable to the one attained by the free GOx and HRP enzyme mixture, although in this case, the reaction occurs faster. The distinct biotinylated fibrils differed slightly in their reaction velocities. This might be caused by a different amount of streptavidin conjugates in the different fibrils or by small differences in the effective concentration of fibrils in the final solution since we do not expect that the identity of the fibrils or their morphology would impact *per se* the activity of the externally added enzymes.

It was recently shown that linking of the two enzymes together or placing them in close proximity does not result in activity enhancement in the GOx-HRP cascade because H_2_O_2_ diffuses rapidly;^67^ therefore, we did not expect our fibrils to be more active than the mixture of the two free streptavidin-enzyme conjugates alone. The higher velocity of this control reaction likely responds to the fact that the concentration of one or both enzymes is higher than those in the fibril-containing solutions. Despite the fact that the concentration of the enzymes in the soluble mixture is the same as the one used in the incubations with the fibrils, not necessarily all the available streptavidin-enzyme would bind to the fibrils. In addition, the control reaction was not subjected to washing steps, which might result in the loss of a proportion of the initially functionalized fibrils.

GOx is the rate-limiting enzyme in this coupled reaction since it displays the lowest reaction velocity.^67^ We set up an experiment incubating the biotinylated scaffolds with streptavidin-HRP alone, washing them up to remove any non-attached HRP and adding soluble GOx to the solution at the same concentration as that in the mixture of the two free enzymes, that is, at the maximum concentration that can be potentially attached to the fibrils (Figure S4a,b). Under these conditions, all the reactions are very efficient and equally fast, indicating that, in the double functionalized fibrils, the velocity of the cascade reaction is determined by the concentration of the slower GOx enzyme. Of course, in the absence of soluble GOx, streptavidin-HRP-conjugated fibrils exhibited a neglectable ability to convert glucose into ABTS^+^ (Figure S4c,d).

Overall, we can conclude that the three biotinylated scaffolds allow the effective immobilization of two different streptavidin-enzyme conjugates to create enzyme cascades. Additionally, a single enzyme can be immobilized on top of the fibrils and the cascade initiated by the addition of the rate-limiting enzyme in its soluble form.

The derivatization of protein fibers with the avidin-biotin chemistry has been previously used to develop a glucose biosensor by modifying the fibers post-assembly with thiol groups and coupling them to a gold surface.^45^ However, only GOx was used as a sensor, and therefore the system lacked the signal amplification characteristic of enzyme cascades that we use here.

### 3.6 Functionalization of Nanofibers with Quantum Dots

**Figure 6:**
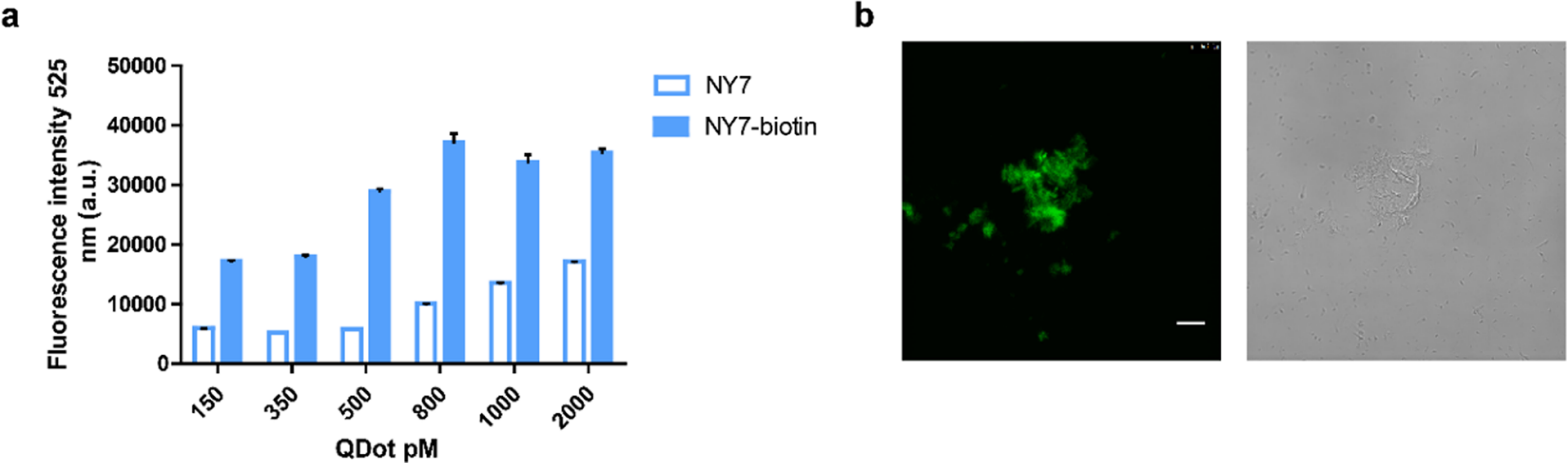
Quantum dots binding to biotinylated amyloid scaffolds. (a) Fluorescence emission intensity of streptavidin-QD bound to NY7 and biotinylated NY7 fibers. (b) Confocal microscopy images of biotinylated NY7 fibers decorated with QD. Left and right images correspond to fluorescence and bright-field microscopy, respectively. The scale bar corresponds to 10 μm.

Finally, we addressed the possibility of modifying the heptapeptide fibers with optical probes. To this aim, biotinylated scaffolds were decorated with the inorganic fluorophores’ quantum dots (QD), whose particular semiconducting properties make them suitable for a wide variety of applications. The binding of streptavidin-QD was confirmed by incubating biotinylated NY7 fibers, used as a model system, with QD at concentrations ranging from 150 to 2000 pM and measuring the fluorescence emission at 525 nm. The specificity of the binding reaction was tested using nonbiotinylated NY7 fibers as controls. Under the assay conditions, the fluorescence signal in biotinylated NY7 fibers increased linearly with the concentration of QD up to 800 pM when the signal became saturated (Figure 6a). A certain concentration-dependent nonspecific binding of streptavidin-QD to control fibers was observed. However, under all conditions, the fluorescence emitted by modified fibers was significantly higher than those on nonbiotinylated ones, with a fourfold difference in the emitted signal at 800 pM. Biotinylated amyloid scaffolds decorated with QD appeared as highly fluorescent when visualized by confocal microscopy (Figure 6b).

The possibility to accommodate QD would allow endorsing biotinylated amyloid scaffolds with fluorescent and semiconductor properties.

## 4 Conclusions

The decoration of nanostructures with active proteins such as enzymes, fluorescent proteins, or antibodies and chemical entities, like metallic or fluorescent nanoparticles and other ligands, is of major relevance for their nanotechnological use.

Here, we introduce a modular and simple method for functionalizing peptide nanofibers based on the extraordinarily high-affinity interaction between the streptavidin protein and biotin. The incorporation of the biotin moieties to the nanofibers occurs during assembly and therefore does not require the post-assembly modification of fibrillar matrices reported in previous studies.^43−45^ Moreover, due to their reduced size, both nonbiotinylated and biotinylated peptides can be commercially acquired with high purity at low cost. The molecular architecture of prion-inspired fibrils,^22^ their slow and hierarchal assembly, and the doping strategy result in functionalized fibrils with a defined biotin ratio, in contrast to the uncertain number of biotins attached in post-assembly modification strategies.^44,45^ In addition, it facilitates that consecutive biotinylated peptides become distanced enough to avoid steric interference between incoming tetrameric streptavidin units, which, as shown, results in highly functionalized fibrils.

**Figure 7:**
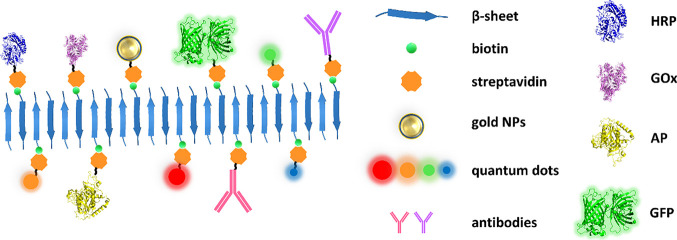
Schematic representation of biotinylated functionalized heptapeptide scaffolds. In the scheme, there are some examples of functionalization shown using the biotin-streptavidin system. This strategy allows the functionalization of these scaffolds with a huge range of possibilities, including different enzymes (HRP, GOx, or PA), fluorescent proteins (GFP), quantum dots, antibodies, or gold NPs, among many others.

A large number of hydrogen bonds at the interface of the steric zippers formed by prion-inspired peptides^22^ results in highly stable matrices whose chemical and physical resistance can be further improved, if required, through UV light-induced Tyr cross-linking.^21^ This property, together with the ability to bind and retain metal nanoparticles, quantum dots, or enzymes like HRP, AP, or GOx and, potentially, any streptavidin-conjugate of interest (Figure 7), suggests that they can find application in plasmon resonance techniques, buildup of hybrid protein–metal nanowires, imaging uses, or as recyclable biocatalysts. All these applications might be implemented with the anchorage of a single type of chemical or biological entity. To the best of our knowledge, we show for the first time a streptavidin-biotin amyloid-like scaffold with simultaneous decoration of its surface with two different sensing proteins, a property that allows sustaining coupled enzymatic reactions to detect different substrates and ligands, as we show here implementing a glucose biosensor using commercially available streptavidin conjugates.

Multidecorated fibril assemblies, similar to the ones that we describe here, might find application as bio-nanoplatforms to develop tunable biosensors to detect a great diversity of molecules, just playing with the nature and ratio of the enzymes fused to streptavidin.

Taken together, we describe here a simple and cost-effective method to generate a modular, biocompatible, biotin-containing, amyloid-like nanostructured system that can be easily functionalized with any streptavidin conjugate of interest. The diversity of commercially available streptavidin derivatives, from metals to antibodies or nucleic acids, suggests that these assemblies might find application in a range of different nanotechnologies.

## Supporting Information Available

Critical aggregation concentration assessment for nonbiotinylated and biotinylated peptides, physicochemical characterization of nonbiotinylated self-assembled peptides, dot blot detection of streptavidin-AP conjugates bound to biotinylated assemblies, activity of biotinylated peptides in the presence and absence of soluble GOx, CAC values calculated from the linear regression, FTIR secondary structure content of nonbiotinylated and biotinylated scaffolds, and quantification of gold NP retention by nonbiotinylated and biotinylated peptides (PDF)

## Abbreviations

ABTS: 2,2′-azino-bis(3-ethylbenzothiazoline-6-sulfonic acid)
AP: alkaline phosphatase
ICP-OES: inductively coupled plasma-atomic optic emission spectrometry
GOx: glucose oxidase
HRP: horse radish peroxidase
NPs: nanoparticles
pNP: *p*-nitrophenol
pNPP: *p*-nitrophenyl phosphate
PVDF: polyvinylidene difluoride
QD: quantum dots
TEM: transmission electron microscopy
Th-T: thioflavin-T
TTBS: Tween20 Tris buffered saline
