# Supplementary material for: Functionalized Prion-Inspired Amyloids for Biosensor Applications
Source: Biomacromolecules. 2021 Jul 1;22(7):2822–33. doi: 10.1021/acs.biomac.1c00222 (PMC8483438; doi:10.1021/acs.biomac.1c00222)
Supplement: Supplementary file 1 — bm1c00222_si_001.pdf [file bm1c00222_si_001.pdf]

Supplementary Information

Functionalized Prion-Inspired Amyloids for Biosensors Applications

*Marta Díaz-Caballero‡, Susanna Navarro‡ and Salvador Ventura**

Institut de Biotecnologia i de Biomedicina and Departament de Bioquímica i Biologia Molecular; Universitat Autònoma de Barcelona; 08193 Bellaterra (Barcelona), Spain.

E-mail: salvador.ventura@uab.es

‡ These authors contributed equally

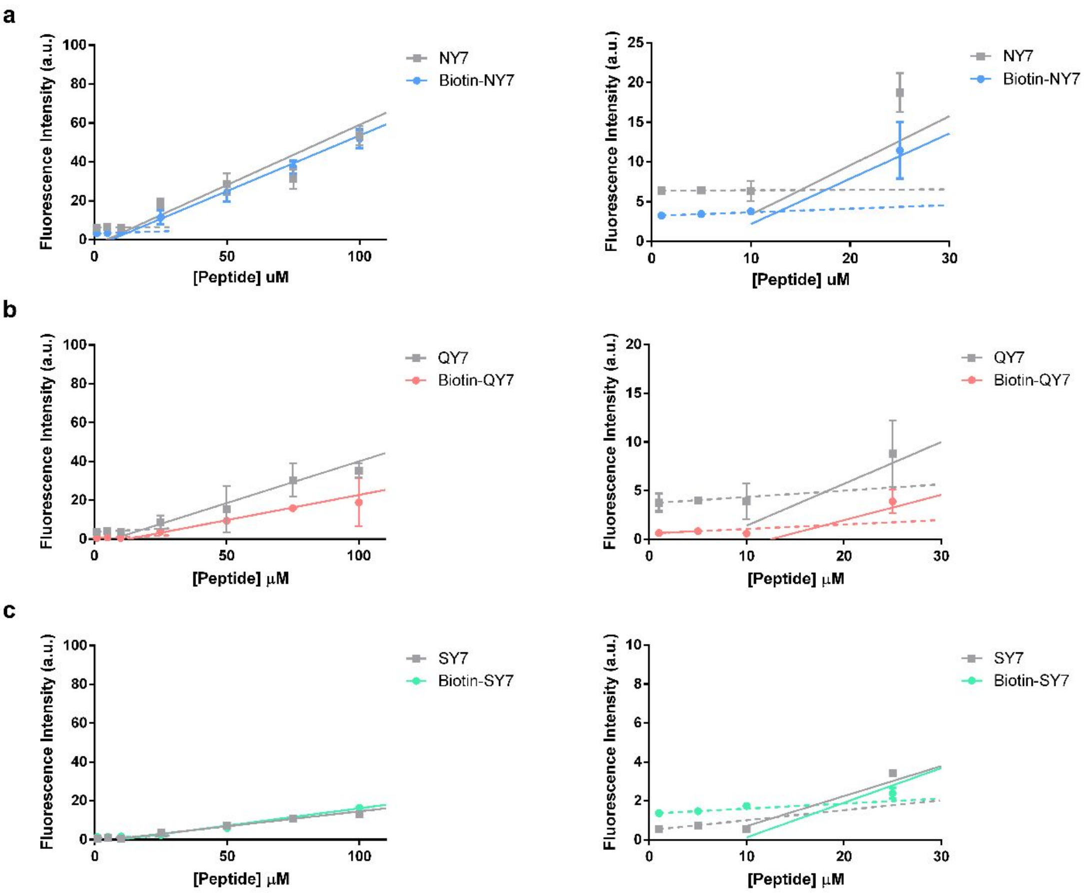


**Figure S1**. Critical aggregation concentration (CAC) assay for non-biotinylated and biotinylated NY7, QY7 and SY7 peptides. Self-assembly of non-biotinylated (grey lines) and biotinylated a) NY7 (blue), b) QY7 (red) and c) SY7 peptides (green) was assessed using Th-T fluorescence signal, and the concentration of peptide *versus* the Th-T signal is plotted. Continuous lines correspond to linear fitting of Th-T signal and discontinuous lines to the fitting at the low concentration points (from 0 to 10 µM) which display basal Th-T signal. Right column graphs correspond to a magnification of the respective left panels for visualization of the linear regression at low concentrations. The CAC value was estimated from the intersection of the two linear regression lines. Bars correspond to SD from three independent replicates.

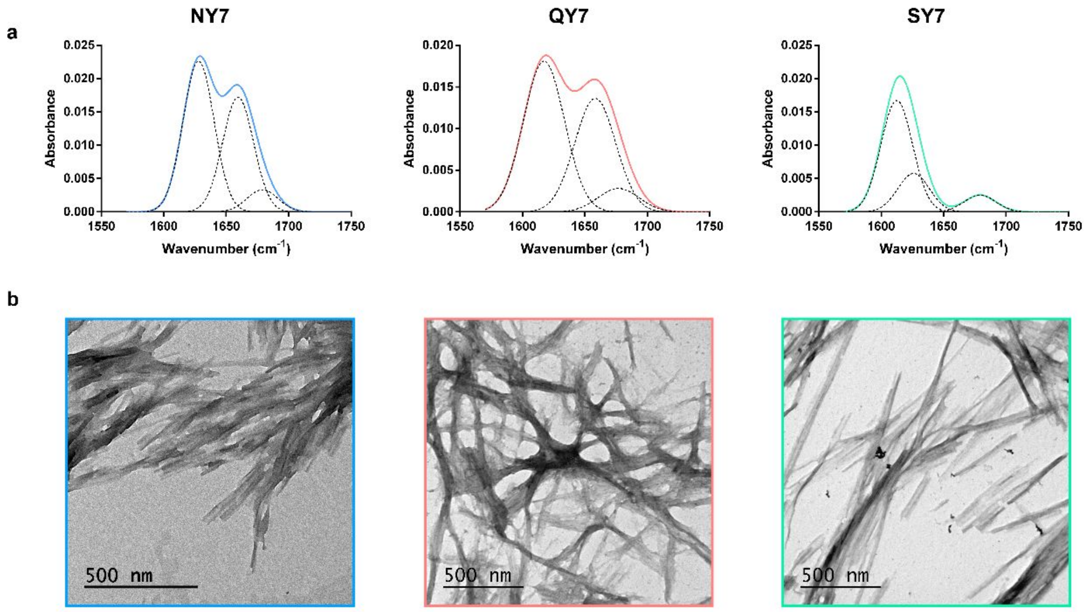


**Figure S2**. Physicochemical characterization of non-biotinylated self-assembled peptides. a) FTIR absorbance spectra in the amide I region (solid line) of non-biotinylated NY7 (blue), QY7 (red) and SY7 (green) assembled peptides. Dashed black lines indicate the different signals contributing to the spectrum. b) TEM images of the self-assembled fibrils. Scale bar corresponds to 500 nm.

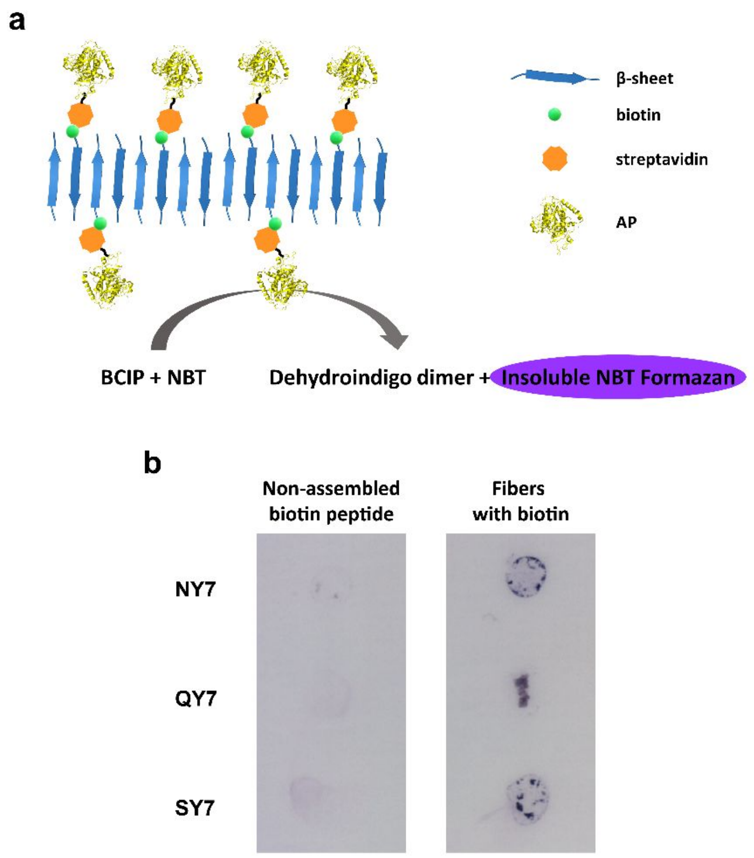


Figure S3. Detection of streptavidin-AP conjugate activity bound to biotinylated-NY7, biotinylated-QY7 and, biotinylated-SY7 amyloid scaffolds. a) Schematic representation of the reaction catalyzed by AP within the scaffold. b) Dot blot of the biotinylated-NY7, biotinylated-QY7 and biotinylated-SY7 scaffolds. Controls correspond to non-assembled biotinylated-peptides.

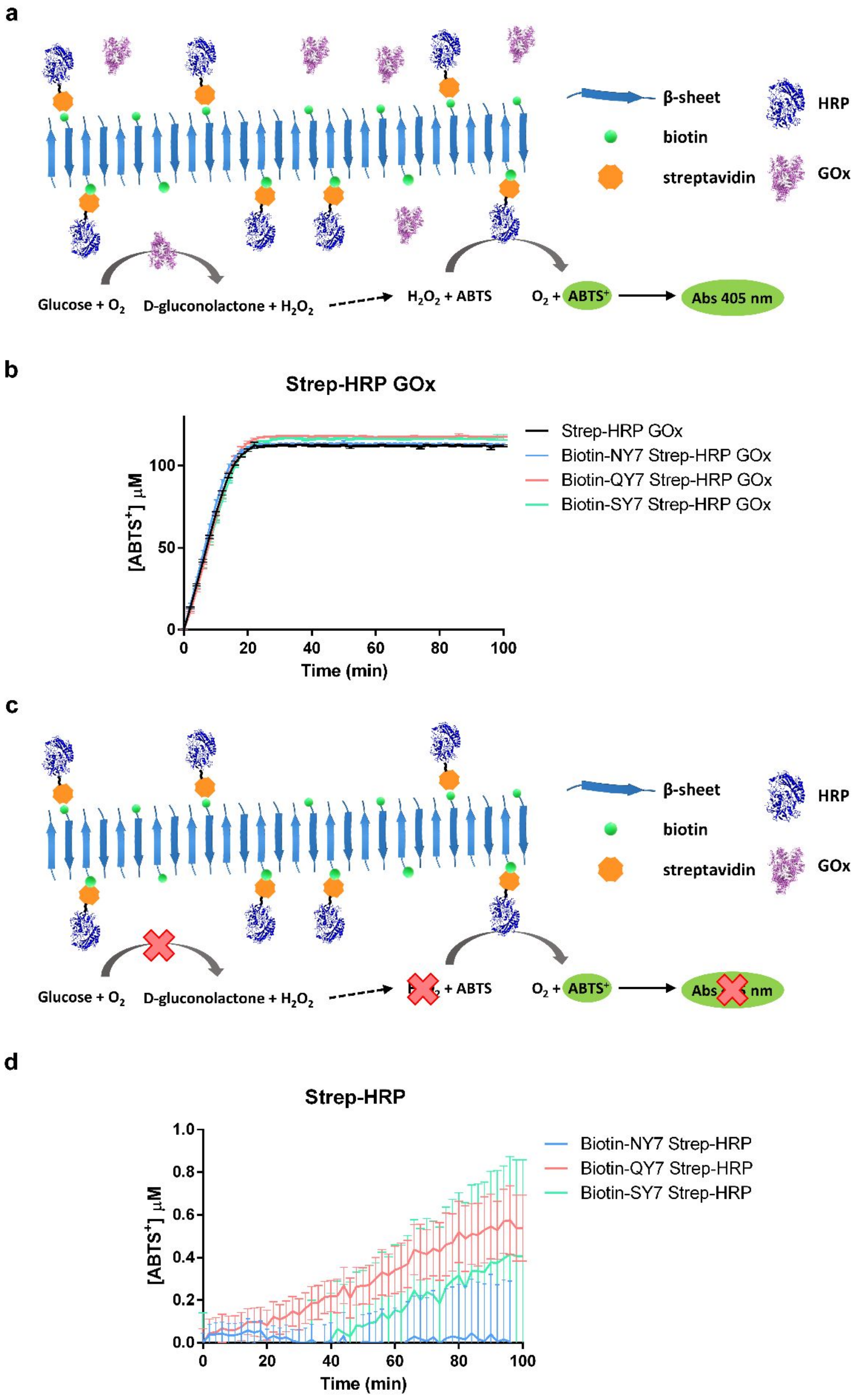
a
β-sheet
HRP
biotin
streptavidin
GOx
Glucose + O2
D-gluconolactone + H2O2
H2O2 + ABTS
O2 + ABTS+
Abs 405 nm
b
Strep-HRP GOx
[ABTS+] μM
Time (min)
Strep-HRP GOx
Biotin-NY7 Strep-HRP GOx
Biotin-QY7 Strep-HRP GOx
Biotin-SY7 Strep-HRP GOx
c
β-sheet
HRP
biotin
streptavidin
GOx
Glucose + O2
D-gluconolactone + H2O2
+ ABTS
O2 + ABTS+
d
Strep-HRP
[ABTS+] μM
Time (min)
Biotin-NY7 Strep-HRP
Biotin-QY7 Strep-HRP
Biotin-SY7 Strep-HRP

**Figure S4**. Activity of biotinylated-NY7, biotinylated-QY7 and biotinylated-SY7 amyloid scaffolds functionalized with streptavidin-HRP in the presence or absence of soluble GOx. In (a) and (c) it is schematically represented the reaction occurring in the graph below. (b) Biotinylated peptides in the presence of streptavidin-HRP and soluble GOx. The reaction control (black line) corresponds to a mixture of soluble GOx and soluble streptavidin-HRP (1:1 molar ratio). (d) Biotinylated peptides incubated with streptavidin-HRP in the absence of GOx. Results correspond to the mean of three independent experiments ± standard deviation.

**Table S1**. CAC values for non-biotinylated and biotinylated NY7, QY7 and SY7 peptides. Values were obtained from the linear regressions in the graphs shown in Figure S1.

| Peptide | Non-Biotinylated | Biotinylated |
|---|---|---|
| NY7 | 14.98 μM | 12.83 μM |
| QY7 | 16.54 μM | 17.34 μM |
| SY7 | 12.99 μM | 19.73 μM |

**Table S2**. FTIR secondary structure content. Percentage of the absorbance area in the amide I region of FTIR spectra assigned to the secondary structure components of non-biotinylated and biotinylated NY7, QY7 and SY7 fibrils. The β-sheet contribution is indicated in bold. Percentages were obtained from the fittings shown in Figure 1c and Figure S2a.

| ASSIGNMENTS | | Intermolecular β-sheet 1615 – 1636 $cm^{-1}$ | Disordered/ Loops/Turns 1658 – 1662 $cm^{-1}$ | β-sheet 1675 – 1682 $cm^{-1}$ |
|---|---|---|---|---|
| NY7 | No-biotin | **52.41** | 39.86 | **7.74** |
| | biotin | **53.94** | 37.53 | **8.53** |
| QY7 | No-biotin | **52.40** | 39.43 | **8.17** |
| | biotin | **41.08** | 45.45 | **13.46** |
| SY7 | No-biotin | **94.19** | - | **5.81** |
| | biotin | **86.82** | - | **13.18** |

**Table S3**. Quantification of gold-NPs content in non-biotinylated and biotinylated NY7, QY7 and SY7 fibrils measured by ICP-OES.

| ng Gold-NP/μg fibrils | Non-Biotinylated | Biotinylated |
|---|---|---|
| **NY7** | 9.68 ± 1.10 | 33.48 ± 1.11 |
| **QY7** | 15.09 ± 0.10 | 35.52 ± 0.67 |
| **SY7** | 18.45 ± 3.48 | 45.36 ± 1.05 |
